# Aromatase Inhibition Reduces Insulin Sensitivity in Healthy Men

**DOI:** 10.1210/jc.2015-4146

**Published:** 2016-03-11

**Authors:** Fraser W. Gibb, Natalie Z. M. Homer, Abdullah M. M. Faqehi, Rita Upreti, Dawn E. Livingstone, Kerry J. McInnes, Ruth Andrew, Brian R. Walker

**Affiliations:** British Heart Foundation Centre for Cardiovascular Science, University of Edinburgh, Queen's Medical Research Institute, Edinburgh EH16 4TJ, United Kingdom

## Abstract

**Context:**

Deficiency of aromatase, the enzyme that catalyzes the conversion of androgens to estrogens, is associated with insulin resistance in humans and mice.

**Objective:**

We hypothesized that pharmacological aromatase inhibition results in peripheral insulin resistance in humans.

**Design:**

This was a double-blind, randomized, controlled, crossover study.

**Setting:**

The study was conducted at a clinical research facility.

**Participants:**

Seventeen healthy male volunteers (18–50 y) participated in the study.

**Intervention:**

The intervention included oral anastrozole (1 mg daily) and placebo, each for 6 weeks with a 2-week washout period.

**Main Outcome Measure:**

Glucose disposal and rates of lipolysis were measured during a stepwise hyperinsulinemic euglycemic clamp. Data are mean (SEM).

**Results:**

Anastrozole therapy resulted in significant estradiol suppression (59.9 ± 3.6 vs 102.0 ± 5.7 pmol/L, *P* = < .001) and a more modest elevation of total T (25.8 ± 1.2 vs 21.4 ± 0.7 nmol/L, *P* = .003). Glucose infusion rate, during the low-dose insulin infusion, was lower after anastrozole administration (12.16 ± 1.33 vs 14.15 ± 1.55 μmol/kg·min, *P* = .024). No differences in hepatic glucose production or rate of lipolysis were observed.

**Conclusion:**

Aromatase inhibition reduces insulin sensitivity, with respect to peripheral glucose disposal, in healthy men. Local generation and action of estradiol, at the level of skeletal muscle, is likely to be an important determinant of insulin sensitivity.

## Abstract (precis)

Aromatase inhibition reduces insulin sensitivity, with respect to peripheral glucose disposal, in healthy men. Local generation and action of estradiol, at the level of skeletal muscle, is likely to be an important determinant of insulin sensitivity.

Although best known for their role in reproduction, both androgens and estrogens exert metabolic effects (1). In men, T deficiency is associated with an increased risk of type 2 diabetes mellitus (2) and pharmacological androgen deprivation, used in the treatment of prostate cancer, is associated with deteriorating insulin sensitivity (3). There is inconsistent evidence that testosterone replacement therapy improves insulin sensitivity in hypogonadal men (4, 5).

In postmenopausal women, estrogen replacement reduces the risk of type 2 diabetes mellitus (6). Estrogens are generated from substrate androgens through the action of the cytochrome P450 enzyme aromatase (7). The circulating concentration of estrogens may be much less important than local tissue generation, particularly in men and postmenopausal women, in whom the local activity of aromatase in skeletal muscle and adipose tissue is likely to account for most estrogen production and action (8). It is possible that the metabolic phenotype in androgen-deficient men is largely a consequence of downstream estrogen deficiency.

Supporting this hypothesis, male aromatase knockout mice have increased adiposity (9), hepatic steatosis (10), and insulin resistance (11), with similar features observed in rare cases of human aromatase deficiency (12); in both cases, estrogen replacement largely reverses the abnormal phenotype. In healthy male volunteers, anastrozole has been shown to increase adiposity, particularly in the intraabdominal compartment (13). Aromatase inhibitors are widely used for long-term treatment in breast cancer, but any effect on fuel metabolism has not been addressed.

We hypothesized that pharmacological aromatase inhibition induces insulin resistance in healthy male volunteers. To test this hypothesis, we performed a randomized, placebo-controlled, crossover study using the aromatase inhibitor anastrozole, assessing insulin sensitivity with gold-standard stable isotope tracer methodology.

## Subjects and Methods

### Study design

This was a double-blind, randomized, placebo-controlled, balanced crossover study in healthy male volunteers. Ethical approval was obtained from the Lothian Research Ethics Committee and informed written consent was obtained from participants.

After a screening visit, participants received 6 weeks of anastrozole (1 mg daily; Astra Zeneca) and 6 weeks of placebo, administered in identical capsules (Tayside Pharmaceuticals), in random order with a 2-week washout period.

### Participants

Participants were recruited through newspaper and poster advertisements. Inclusion criteria were men aged 18–65 years with normal screening blood tests (urea and electrolytes, liver function tests, lipid profile, thyroid function tests, and full blood count). Exclusion criteria were any significant current illness, use of regular medication, alcohol excess (defined as > 28 U/wk) and an inability to give informed consent. All screened volunteers (n = 20) fulfilled these criteria, although three individuals elected not to proceed to randomization. Adherence was assessed by the presence of anastrozole in plasma and suppression of circulating estrogens in the active phase.

### Outcomes

The primary outcome measure was insulin sensitivity as assessed by glucose disposal during a hyperinsulinemic-euglycemic clamp (14). Additional end points included rates of endogenous glucose production and lipolysis, body fat, lipid profile, plasma adipocytokines, and mRNA transcript abundance in sc adipose tissue.

### Clinical methods

Participants attended the Clinical Research Facility at 7:30 am after an overnight fast on two occasions (at completion of each 6 wk course of tablets). Height, weight, and blood pressure (BP) (seated) were measured. Body fat was assessed with an OMRON BF306 bioimpedance device (OMRON Healthcare). A three-phase hyperinsulinemic-euglycemic clamp study was conducted with infusion of d2-glucose (6,6-^2^H_2_-glucose) and d5-glycerol (1,1,2,3,3,-^2^H_5_-glycerol) tracers over 270 minutes. From 0 to 90 minutes, only stable isotope tracers were infused, with initial priming doses of d2-glucose (17 μmol/kg) and d5-glycerol (1.6 μmol/kg) over 1 minute, followed by a continuous infusion of d2-glucose (0.22 μmol/kg·min) and d5-glycerol (0.11 μmol/kg·min). Tracer infusion rates were maintained for the remainder of the clamp study. From 90 to 180 minutes, low-dose insulin (Actrapid; Novo Nordisk; 10 mU/m^2^·min) was infused to assess suppression of lipolysis and endogenous glucose production. From 180 to 270 minutes, high-dose insulin was infused (40 mU/m^2^·min) to assess peripheral glucose disposal. Between 90 and 270 minutes, 20% dextrose (Baxter) was infused to maintain euglycemia (4.5–5.5 mM) as determined by the measurement of arterialized blood samples every 5 minutes (Accu-Chek Advantage; Roche). Arterialized venous blood samples were obtained at baseline and as four steady-state samples taken over 15 minutes at the end of each phase of the clamp study.

Biopsies of sc, periumbilical adipose tissue were taken 2 days in advance of the clamp studies using a 14-gauge needle, under local anesthesia, and frozen immediately on dry ice.

### Laboratory methods

Glucose, urea and electrolytes, thyroid and liver function, and lipid profile were measured by autoanalyzer (Architect c16000 analyzer; Abbott Diagnostics Ltd). SHBG was measured by an enzyme-linked immunoassay (Demeditec Diagnostics); plasma insulin by ultrasensitive ELISA (DRG); LH by an in-house ELISA (Supplemental Material); plasma nonesterified fatty acids (NEFAs) by a coupled enzyme reaction assay (Zen-Bio Inc); and plasma leptin, monocyte chemoattractant protein 1, IL-8, adiponectin, and resistin by a Milliplex immunoassay (Merck Millipore). Plasma glucose, d2-glucose, glycerol, and d5-glycerol were quantified by gas chromatography/mass spectrometry (15). mRNA abundance of transcripts of interest (Supplemental Table 1) in sc abdominal adipose tissue was assessed by real-time quantitative PCR (16) and presented in relation to that of the housekeeping gene *cyclophylin A*, which did not differ between the groups and is not known to be influenced by sex steroid hormones.

Plasma T and androstenedione were quantified by liquid chromatography and tandem mass spectrometry (LC-MS/MS) (17), adapted for smaller volume of plasma (30 μL), and using Oasis HLB 10 mg cartridges (Waters). Estradiol and estrone were quantified by LC-MS/MS (18). Bioavailable and free T were calculated as previously described (19). Plasma anastrozole was quantified by LC-MS/MS (Supplemental Material).

### Tracer kinetic calculations

Steady state of tracer to tracee ratios (TTRs) was tested by Spearman's correlation of time vs TTRs of the four measurements during each steady-state period, and steady state was accepted when *P* > .10. Tracer kinetics were calculated using mean values from the relevant steady-state period of the hyperinsulinemic-euglycemic clamp. M value = glucose infusion rate at steady state; rate of disposal (Rd) of glucose = d2-glucose infusion rate to TTR; endogenous glucose production = Rd glucose − glucose infusion rate; and rate of appearance (Ra) of glycerol = d5-glycerol infusion rate to TTR. Adjustments were made to account for naturally occurring mass+2 glucose.

### Sample size and statistical analysis

Based on previously published data (20), a sample size of 20 was calculated to have 80% power to detect a 15% difference in glucose disposal to *P* < .05. Due to difficulties in recruitment, only 17 participants completed the study. All statistical analyses were performed using SPSS for Windows, version 19 (IBM). Data are presented as mean (SEM). Comparisons between the anastrozole and placebo treatment periods were assessed with paired Student's *t* tests. When data were not normally distributed, logarithmic transformation was undertaken. When transformation did not result in normally distributed data, a related-samples Wilcoxon signed rank test was used.

## Results

### Participant characteristics

Seventeen individuals entered the study, all of whom completed the protocol with no significant adverse events reported. The first treatment phase was anastrozole in nine subjects and placebo in the remaining eight. Six participants consented to paired sc adipose biopsies at the completion of each treatment period. The mean age of volunteers was 27.7 ± 2.5 years (range 18–50 y).

Anastrozole was detected in plasma from all participants at the end of the active phase, ranging from 18.4 to 197.5 ng/mL (mean 59.9 ± 3.6 ng/mL) and not detected at the end of the placebo phase. One individual had very high anastrozole levels, perhaps due to poor compliance with therapy, so that without this subject anastrozole levels ranged from 18.4 to 93.6 ng/mL and mean 50.8 ± 5.1 ng/mL, but the results were not substantially affected if this participant was omitted from analysis, and there were no correlations between anastrozole concentrations and changes in insulin sensitivity.

### Effects of anastrozole on insulin sensitivity

**Figure 1.**
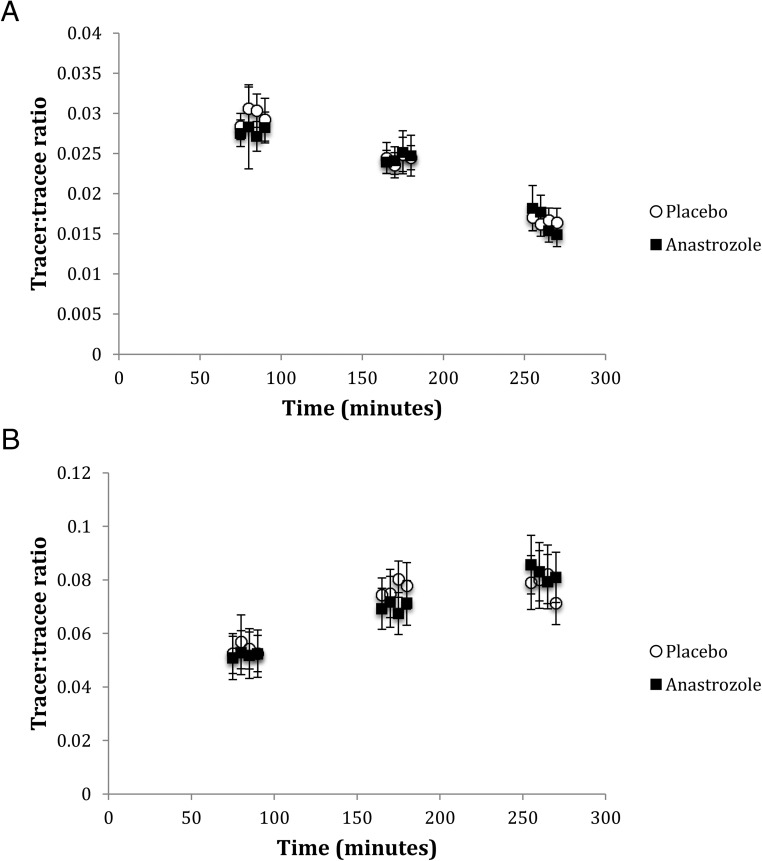
TTR for glucose (A) and glycerol (B) across the hyperinsulinemic clamp.

Plasma glucose concentrations were stable (Supplemental Figure 1), and steady-state d2-glucose enrichments were achieved during each phase of the hyperinsulinemic euglycemic clamp (Figure 1), although glucose TTRs fell modestly during low-dose insulin infusion (−0.004 ± 0.001, *P* < .001) and substantially during high-dose insulin infusion (−0.012 ± 0.001, *P* < .001). Predicted suppression of endogenous glucose production, inhibition of lipolysis, and stimulation of peripheral glucose disposal by insulin were observed (Tables 1 and 2).

**Table 1.** Effect of Anastrozole on Indices of Insulin Sensitivity for Glucose Metabolism.

	Anastrozole	Placebo	*P* Value
During tracer without insulin infusion			
Glucose, mM	5.7 (0.3)	5.7 (0.2)	.870
Insulin, mU/L	5.8 (0.8)	5.8 (1.0)	.988
Endogenous glucose production, μmol/kg·min	7.77 (0.46)	7.50 (0.46)	.271
During low-dose insulin infusion			
Glucose, mM	6.5 (0.4)	6.5 (0.3)	.769
Insulin, mU/L	15.1 (1.2)	16.7 (1.8)	.378
EGP, μmol/kg·min	3.79 (1.01)	3.37 (1.10)	.562
Suppression of endogenous glucose production, %	61.2 (13.0)	65.0 (12.5)	.723^a^
M value, μmol/kg·min	12.16 (1.33)	14.15 (1.55)	.024
Rd glucose, μmol/kg·min	15.04 (1.13)	16.46 (1.50)	.044^a^
During high dose insulin infusion			
Glucose, mM	5.7 (0.2)	5.8 (0.2)	.651
Insulin, mU/L	48.1 (3.7)	53.6 (4.4)	.298
M value, μmol/kg·min	41.80 (3.66)	43.13 (4.16)	.599

Abbreviation: EGP, endogenous glucose production. Data are mean (SEM).

a Compared by related-samples Wilcoxon signed rank test.

**Table 2.** Effects of Anastrozole on Lipid Profile and insulin Sensitivity Indices for Lipolysis

	Anastrozole	Placebo	*P* Value
Fasting before infusion			
Total cholesterol, mM	3.86 (0.13)	4.12 (0.14)	.041
LDL-cholesterol, mM	2.39 (0.14)	2.56 (0.14)	.118
HDL-cholesterol, mM	1.01 (0.05)	1.09 (0.05)	.080
Triglycerides, mM	1.02 (0.09)	1.05 (0.16)	.770
NEFAs, μM	1011.6 (88.2)	982.4 (87.6)	.793
During tracer infusion without insulin infusion			
Glycerol, μM	62.3 (5.1)	66.4 (4.7)	.223
Ra glycerol, μmol/kg·min	2.85 (0.36)	2.79 (0.34)	.777
NEFAs, μM	1084.0 (71.0)	1051.4 (104.3)	.785
During low-dose insulin infusion			
Glycerol, μM	35.5 (5.2)	38.1 (4.0)	.388
Ra glycerol, μmol/kg·min	1.87 (0.19)	1.73 (0.17)	.287
NEFAs, μmol/L	399.1 (43.3)	294.6 (35.4)	.059
Suppression glycerol from baseline, %	52.8 (4.3)	57.3 (3.4)	.426
Suppression Ra glycerol from baseline, %	28.0 (4.6)	32.0 (4.5)	.302
Suppression NEFAs from baseline, %	61.8 (0.04)	68.7 (0.04)	.087
During high-dose insulin infusion			
Glycerol, μM	27.9 (5.0)	32.1 (3.9)	.086
Ra glycerol, μmol/kg·min	1.69 (0.18)	1.77 (0.20)	.525
NEFAs, μM	173.8 (18.9)	153.7 (24.8)	.437

Abbreviation: HDL, high-density lipoprotein. Data are mean (SEM).

Anastrozole had no effect upon endogenous glucose production or insulin-mediated suppression of endogenous glucose production (Table 1). During low-dose insulin infusion, a significantly lower glucose infusion rate (M value) was required to maintain euglycemia and Rd glucose tended to decrease after the anastrozole administration (Table 1). During the high-dose insulin infusion, no significant difference in the M value was observed (Table 1).

Lipolysis, as determined by Ra glycerol, was not influenced by anastrozole, although nonsignificant trends toward higher plasma NEFA concentration and the attenuation of insulin-mediated suppression of NEFAs were observed during low-dose insulin infusion (Table 2).

### Effects of anastrozole on BP, body composition, and lipids

Anastrozole administration was associated with a 4-mm Hg increase in systolic BP compared with placebo (138 ± 3 vs 134 ± 3 mm Hg, *P* < .05) although no significant difference was observed in diastolic BP (79 ± 2 vs 78 ± 2 mm Hg, *P* = .847). Heart rate was correspondingly significantly lower during anastrozole treatment (66 ± 3 vs 71 ± 3 bpm, *P* < .05). No significant differences in weight (82.2 ± 3.4 vs 81.8 ± 3.4 kg, *P* = .404), body mass index (25.9 ± 1.1 vs 25.7 ± 1.1 kg/m^2^, *P* = .445), or percentage body fat (16.4% ± 1.9% vs 16.4% ± 1.9%, *P* = .957) were observed between anastrozole and placebo treatment. Plasma leptin was significantly lower after anastrozole (4249 ± 999 vs 5890 ± 1349 pg/mL, *P* = .039), although no difference was observed in adiponectin, resistin, IL-8, or monocyte chemotactic protein-1 (Supplemental Table 2). Total cholesterol was lower during the anastrozole phase (3.86 ± 0.13 vs 4.12 ± 0.14 mmol/L, *P* = .041), as a consequence of nonsignificant trends toward lower high-density lipoprotein cholesterol (7.4%, *P* = .08) and low-density lipoprotein (LDL) cholesterol (6.6%, *P* = .12) (Table 2). Aromatase inhibition exerted only minimal effects on sc adipose mRNA transcript expression. Of 27 genes assessed (Supplemental Table 3), only the down-regulation of the estrogen receptor β (45.1%, *P* = .04) and perilipin 2 (8.8%, *P* = .045) were statistically significant.

### Effects of anastrozole on sex steroid concentrations

Aromatase inhibition resulted in a 20.5% increase in plasma total T (*P* = .003), a 41.3% decline in estradiol (*P* < .001), a 43.6% (*P* < .001) fall in estrone, and a compensatory 31.4% rise in LH (*P* < .001) (Table 3). These pharmacodynamic changes did not correlate with changes in insulin sensitivity (not shown).

**Table 3.** Effect of Anastrozole on Sex Hormones in Plasma

	Anastrozole	Placebo	*P* Value
T, nmol/L	25.8 (1.2)	21.4 (0.7)	.003
Bioavailable T, nmol/L	17.2 (1.0)	13.8 (0.7)	<.001
Calculated free T, pmol/L	858 (51)	671 (36)	<.001
Androstenedione, nmol/L	3.49 (0.10)	2.97 (0.12)	.002
Estradiol, pmol/L	59.9 (3.63)	102.0 (5.65)	<.001
Estrone, pmol/L	71.1 (4.07)	126.1 (4.74)	<.001
SHBG, nmol/L	19.1 (1.5)	20.4 (1.5)	.06
LH, IU/L	3.5 (0.3)	2.4 (0.3)	<.001

Data are mean (SEM).

None of the effects of anastrozole were explained by an order effect because all were independent the order of drug vs placebo administration (data not shown).

## Discussion

In a randomized, double-blind, placebo-controlled, cross-over study, we have shown that aromatase inhibition with anastrozole impairs insulin-stimulated peripheral glucose disposal, without affecting hepatic glucose production or lipolysis. An effect on the primary end point for the study was achieved despite recruiting 17 of a target 20 participants.

Previous investigators assessed effects of letrozole administered for 4 weeks in 18 healthy men (divided into younger and older age groups) (21) and reported improved insulin sensitivity, inferred from changes in fasting glucose and insulin, but only in younger subjects. A subsequent study by the same investigators assessed letrozole effects over a 1-week period either alone or in combination with transdermal estradiol (22); hyperinsulinemic euglycemic clamp studies suggested improved insulin sensitivity in the letrozole group (although only after correction for fat free mass) but not in the group receiving concomitant estradiol replacement. However, it is difficult to conclude that estradiol suppression is metabolically advantageous in men because supraphysiological estradiol replacement resulted in LH suppression and consequently subnormal T concentrations. Furthermore, letrozole has been associated with significant, sustained reductions in both morning plasma cortisol (23) and ACTH-stimulated cortisol (24), an effect that is not observed with anastrozole (25). It is not therefore possible to exclude an effect on glucocorticoid signaling as a mediator of any effect observed with letrozole. No previous study of the metabolic effects of aromatase inhibitors has used gold-standard stable isotope tracer euglycemic hyperinsulinemic clamp methodology. Other studies relying upon fasting glucose and insulin measurements have failed to demonstrate an effect of aromatase inhibition upon insulin sensitivity (26–28), but this may reflect the lower sensitivity of the methodology.

We found a significant effect of anastrozole to suppress glucose disposal during low-dose insulin infusion, measured both by M value (glucose infusion rate) and Rd glucose (tracer kinetics). The 14.1% decrease in glucose disposal we observed after a short course of aromatase inhibitor therapy is likely to be of biological significance. We did not detect a difference in M value during high-dose insulin infusion, which may have been a consequence of a particularly insulin sensitive study population in whom ED_50_ effects of insulin may occur at lower concentrations, combined with a predominant effect of anastrozole on ED_50_ rather than maximum insulin response. Moreover, we do not report glucose tracer kinetic data during high-dose insulin infusion because we did not infuse additional D2-glucose tracer in proportion to the increased glucose infusion rate, and, in this insulin-sensitive group, this led to lowering of TTRs and potentially artifactual underestimation of endogenous glucose production (29). Glucose disposal data were also analyzed after the correction for fat-free mass (the primary site of glucose uptake), which did not materially affect the result (data not shown).

The absence of an effect on Ra glycerol suggests that aromatase inhibition does not influence lipolysis in either the fasting or insulin-stimulated state, although the trend for the attenuation of insulin-stimulated plasma NEFA suppression after anastrozole therapy suggests that there could be a small effect that this study was underpowered to detect. Results were similar when the data were adjusted for fat mass as well as body weight (not shown). A lack of effect on lipolysis is supported by the minimal effects observed on adipose tissue mRNA transcript levels for genes involved in the regulation of lipolysis, mirroring earlier observations in aromatase knockout mice (30), albeit the number of paired biopsies we obtained was small and these investigations of secondary end points risk being underpowered. Because insulin stimulates skeletal muscle NEFA uptake (31) and estrogen deprivation in mice reduces the capacity for skeletal muscle fat oxidation (32), it is also possible that any change in NEFA suppression reflects the effects of aromatase inhibition in skeletal muscle.

No differences were observed in body composition as determined by body mass index, weight, or body fat percentage between placebo and anastrozole phases. This is not unexpected after only 6 weeks of therapy. Sixteen weeks of aromatase inhibition therapy in men has been shown to increase body fat, particularly in the intraabdominal compartment, as assessed by sensitive computed tomography and dual-energy x-ray absorptiometry analysis (13). After anastrozole treatment, we observed a 28% reduction in leptin, consistent with the findings of a previous study investigating the effects of letrozole in healthy men (21). Leptin is preferentially secreted by sc rather than omental adipocytes (33), raising the possibility that the observed difference in leptin may represent a shift from sc to visceral adipose deposition. There was no effect of anastrozole on sc adipose leptin mRNA expression, suggesting lower serum leptin concentration is not a consequence of a direct effect on transcription in the sc depot. A subtle shift in fat distribution might indirectly contribute to the observed changes in insulin sensitivity.

Aromatase inhibition in postmenopausal women is associated with profound (>90%) suppression of estradiol (34). However, this is not the case in men, in whom a compensatory increase in LH (and substrate androgens) attenuates the degree of estradiol suppression. Whereas the 41.3% decline in estradiol we observed is consistent with previous reports with letrozole (21, 22, 35), the compensatory rise in T was severalfold lower. Letrozole is more abundant than anastrozole in mouse brain tissue after systemic administration (36), providing a potential explanation for the more modest elevation in LH with anastrozole (31.4% compared with 335%) (35). Notably, this is the first investigation of the metabolic effects of aromatase inhibition to use LC-MS/MS analysis of plasma sex steroid concentrations. The superiority of mass spectrometric analysis has been demonstrated in the context of aromatase inhibition, in which immunometric methods are prone to significantly underestimate estradiol suppression in postmenopausal women (34).

Accordingly, this study with anastrozole is less susceptible than previous studies with letrozole to the criticism that metabolic sequelae are attributable to T excess rather than estrogen deficiency, although we cannot exclude an effect related to the modest (mean 4.4 nmol/L) rise in total T. In men and postmenopausal women, local generation and action of estradiol in adipose tissue and skeletal muscle is likely to be more physiologically relevant than distant action. Plasma concentration of estradiol does not necessarily reflect tissue concentration, as evidenced by the similar breast tissue levels observed in pre- and postmenopausal women, despite markedly divergent plasma concentrations (37). In previous aromatase inhibitor studies in men (21) in whom supraphysiological plasma T concentrations were achieved, the greater delivery of substrate androgens may have limited the desired effect of minimizing local estrogen generation and action in target tissues. It would be desirable to directly assess the effects of aromatase inhibition on target tissue sex steroid hormone concentration.

Anastrozole resulted in a significant reduction in total cholesterol, in contrast to previous studies, which have shown the opposite effect upon LDL cholesterol in healthy younger men (21) or no effect in older men with mild hypogonadism (27). These conflicting findings may reflect differences in the populations studied as well as the less pronounced elevation in plasma T observed in our study.

The rise in systolic BP and lower heart rate observed during aromatase inhibitor treatment was unexpected and contrasts with lower diastolic BP and elevated heart rate as well as reduced baroreflex sensitivity in aromatase knockout mice (38). Estrogens are likely to exert hemodynamic effects at both a central and peripheral level, potentially in a gender-specific manner, demanding a further detailed assessment of the consequences of aromatase inhibition.

In summary, as hypothesized, in healthy men aromatase inhibition resulted in decreased insulin sensitivity, primarily manifest as reduced peripheral glucose disposal. No significant effects on hepatic glucose production or lipolysis were observed. These results suggest suppression of estrogen action in skeletal muscle is the principal mechanism through which aromatase inhibitors exert a deleterious effect on glucose metabolism. This lends support to the hypothesis that, in male androgen deficiency, insulin resistance is largely a consequence of reduced aromatase substrate availability and consequent local estrogen deficiency in target tissue. The metabolic consequences for patients treated with aromatase inhibitors for breast cancer deserve closer investigation.
